# Mismatch type impacts interference and priming activities in the type I–E CRISPR–Cas system

**DOI:** 10.1016/j.jbc.2026.111401

**Published:** 2026-03-25

**Authors:** Phong T. Phan, Meric Ozturk, Elizabeth M. Dougherty, Jerusha Ravishankar, Chaoyou Xue, Dipali G. Sashital

**Affiliations:** Roy J. Carver Department of Biochemistry, Biophysics and Molecular Biology, Iowa State University, Ames, Iowa, USA

**Keywords:** CRISPR–Cas, crRNA, DNA, DNA enzyme, DNA–protein interaction

## Abstract

Type I–E CRISPR–Cas (CRISPR associated) systems direct RNA-guided interference against foreign nucleic acids using the CRISPR RNA (crRNA)–guided Cascade complex and Cas3 helicase–nuclease. DNA targeting by Cascade–Cas3 promotes priming, a mechanism that allows for rapid acquisition of new spacers within the CRISPR array. Target mutations in the protospacer adjacent motif and protospacer adjacent motif–proximal seed region can block interference but may still allow priming. Previous studies have suggested that target mutations to T and A are tolerated but that C and G substitutions are deleterious to interference and priming, respectively. However, the contributions of the crRNA spacer sequence to mutational tolerance remain unclear. Here, we systematically tested the effects of crRNA seed sequences on mutational tolerance. We engineered four *Escherichia coli* strains with variable spacer sequences and tested CRISPR interference and priming against a plasmid library for each strain. Consistent with prior studies, we observe that mutations to C or G in the seed can be highly deleterious, especially at positions 1, 22, and four. However, the corresponding crRNA sequence also strongly impacts the level of defect, with rC–dC and rA/G–dG causing the largest defects in our plasmid library experiments. Using *in vitro* biochemistry, we observe that mismatch type at the first position of the seed affects Cascade conformation and results in reduction in the rates of both Cascade–target binding and Cas3 recruitment. Overall, our results reveal that although nucleotide identity of target mutations is an important determinant of type I–E CRISPR immunity, the crRNA sequence also strongly impacts immune outcomes upon target mutation.

CRISPR–Cas (CRISPR associated) systems are RNA-guided immune systems that allow archaea and bacteria to defend against the constant threat from viruses and other invasive elements (1, 2, 3, 4). During initial infection, a short foreign DNA fragment, called a spacer, is inserted into the CRISPR array of the host chromosome *via* adaptation (1, 5, 6, 7). The CRISPR array is transcribed, and the transcript is processed into mature CRISPR RNAs (crRNAs) (2, 8, 9, 10, 11, 12), each carrying a unique spacer sequence that matches with the invader DNA and associates with Cas proteins to form an effector complex (2, 13, 14). The effector complex uses the crRNA to bind to the complementary target region upon subsequent infection, leading to the destruction of foreign DNA *via* interference (2, 3, 13, 15, 16). In some systems, spacers are preferentially acquired from DNA that is targeted by the Cas effector *via* priming, a positive feedback mechanism that enables rapid adaptation against an invader (17, 18, 19, 20, 21, 22).

*Escherichia coli K12* has a class 1, type I, subtype E (I–E) CRISPR–Cas immune system, which consists of two CRISPR loci and eight *cas* genes located in two operons (Fig. 1*A*) (23). The crRNA effector complex, termed Cascade, comprises a crRNA and an unequal stoichiometry of five Cas proteins, Cas8 (also known as CasA or Cse1), Cas11 (CasB or Cse2), Cas7 (CasC or Cse4), Cas5 (CasD), and Cas6 (CasE or Cse3) (2, 24). In addition, Cas3, a protein containing both a nuclease and helicase domain, is required for target DNA degradation (2, 25, 26, 27, 28, 29).

**Figure 1:**
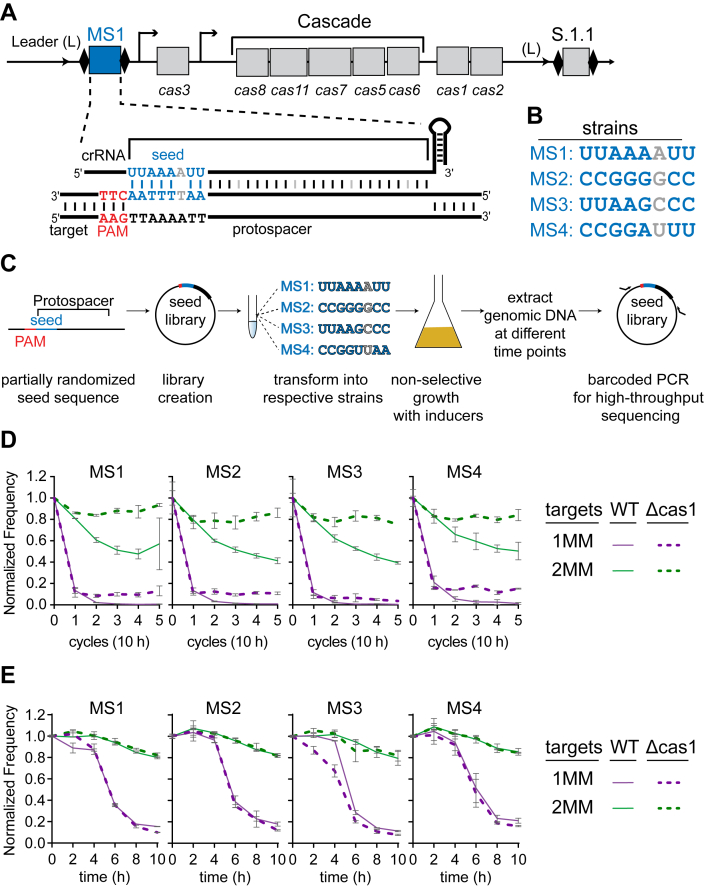
**Time scales of direct interference and priming in an inducible type I–E CRISPR–Cas system.***A*, illustration of the CRISPR–Cas operon from the modified *Escherichia coli* strain used in the study. The strain contains two CRISPRs, each containing two repeats (*black diamonds*) and one spacer (*rectangles* labeled MS1 in CRISPR 22 and S.1.1 in CRISPR 1). The *cas* operon contains the *cas3* gene under the IPTG-inducible *Ptac* promoter and the *cas8*, *cas11*, *cas7*, *cas5*, *cas6*, *cas1*, and *cas2* operons under the arabinose-inducible *araBp8* promoter. Spacer sequences used in this study were introduced into the CRISPR 22 locus (shown in *blue*). A perfect seed target is shown. *B*, sequence of seed sequences for spacer 2.1 for the four different strains. The remainder of the spacer sequence is identical. *C*, workflow of plasmid library selection assay. Four different partially randomized seed sequence libraries were created. Each library was then transformed to corresponding strains and grown for five 10 h cycles or for 10 h with 2 h time points with induction of the *cas* operon and without antibiotic selection. At each time point, the plasmid library was extracted, and the target region was PCR amplified and submitted for high-throughput sequencing. *D* and *E*, overview of seed library sequence selection for WT and Δ*cas1* strains over five 10-h cycles (*D*) and 2 h time points (*E*). Normalized frequency of targets containing one mismatch (1MM, *purple*) and targets containing two MMs (2MM, *green*) over time for WT (*solid line*) and Δ*cas1* (*dashed line*). Fraction of reads for all 1MM or 2MM sequences in each strain were first normalized to the 3–5MM reads (see the Experimental procedures section) and then to the fraction of reads present in the initial library (0 time point). The average of two replicates is shown, and error bars are the standard deviation with propagated error for normalization. Cas, CRISPR associated.

During infection, Cascade locates target sequences in the invader DNA by initially searching for the PAM (protospacer adjacent motif) (30, 31), a 3-base pair (bp), conserved sequence located just downstream of the crRNA-complementary target that is recognized by the Cas8 subunit of Cascade (21, 32, 33) (Fig. 1*A*). PAM recognition triggers DNA unwinding through RNA strand invasion, in which the guide crRNA and complementary strand of the target DNA base pair, and the nontarget strand is displaced (33, 34). Complete formation of this R-loop triggers Cascade conformational changes that enable recruitment and activation of Cas3 (29, 33, 34, 35), which degrades the DNA through a combination of its helicase and nuclease activities (21, 25, 26, 28, 36). During R-loop formation, target unwinding occurs directionally away from the PAM (35). As a result, base pairing between the crRNA and the “seed” sequence of the target, the eight nucleotides just upstream of the PAM, is important to ensure complete R-loop formation (19, 20, 31) (Fig. 1*A*). Mutations within the PAM and/or seed region reduce interference by Cascade–Cas3 but may still enable primed spacer acquisition, resulting in the acquisition of new spacers that drive interference (17, 18).

Although seed mutations are generally detrimental to CRISPR interference, interference efficiency varies depending on the type of mutation and location within the seed (19, 20, 37, 38). Previous studies have found that target mutations to G and C are more deleterious than mutations to A and T and that mutations at position 44 of the seed can be as deleterious as mutations at the first two positions of the seed (20, 38, 39). While these mutations are generally thought to reduce the rate of Cascade–target binding (31, 39), mutations within the seed sequence also affect Cascade conformation, particularly of the Cas8 subunit (40, 41). Because Cas8 directly interacts with Cas3 during interference (27, 29), alternative conformations of Cas8 are thought to negatively impact Cas3 recruitment and activation for DNA degradation (29, 33, 34, 40, 42). Thus, Cas8 conformation, in addition to defects in target binding, may contribute to the ability of Cascade to direct interference against a target.

A previous study suggested that crRNA spacer sequence can impact interference efficiency, with increased guanine/cytosine (G/C) content providing higher efficiency (43). The spacer sequence may also impact mutational tolerance in the seed, as different mismatch (MM) types would be introduced depending on the crRNA sequence (19). However, studies directly comparing the impact of MM type within the seed are limited. To address this limitation, we engineered four different *E. coli* strains expressing varied crRNA seed sequences with otherwise identical spacer sequences. Using these strains, we systematically tested the functionality of G/C-rich or -poor seed sequences using high-throughput screening of a target MM library. Consistent with previous studies, we observe that C or G mutations at specific positions within the seed are more deleterious than others. However, we find that the corresponding ribonucleotide in the crRNA also has a substantial impact on the level of defect when paired with a C or a G mutation. Using complementary *in vitro* approaches, we observe that C and G mutations impact Cas8 conformation more substantially than T mutations, leading to reduced rates of target degradation when Cascade is prebound to the target DNA. Overall, these results indicate that type I–E immune activities are dependent not only on the mutation type in the target but also on the MM with the crRNA and can result in decreased kinetics of both target binding and Cas3 activation.

## Results

### Evaluating the effects of one or 2 MMs for seed sequences with different G/C contents

To systematically evaluate how different MM types within seed sequence influence CRISPR interference and priming against mismatched targets, we created four strains of *E. coli K-12* in which the G/C content of the seed sequence of one spacer was varied, whereas the remainder of the spacer sequence remained constant. The strains were derived from a modified BW25113 strain (44), in which the *cas* genes are under control of inducible promoters (Fig. 1*A*). Each strain expresses two crRNAs, one from each of the two CRISPRs in the *E. coli* genome. The four seed variants were designed as follows: MS1, A/U-rich; MS2, G/C-rich; MS3, A/U in the first four positions and G/C in the last four positions; and MS4, G/C in the first four positions and A/U in the last four positions (Fig. 1*B*). For each strain, a Δ*cas1* deletion was also engineered. Because the Δ*cas1* strain cannot acquire new spacers, comparison of target loss in Δ*cas1* and WT strains allows for the differentiation of target loss through direct interference by the original spacer or target loss through the acquisition of new spacers *via* priming (7, 19).

For each strain, we carried out an *in vivo* plasmid library selection assay followed by high-throughput sequencing (HTS). In this approach, target libraries containing seed sequence MMs were constructed for each strain, with the majority of sequences containing one or two MMs (Figs. 1*C* and S1*A*). The sixth position of the crRNA does not form a base pair with the target DNA, so it was not varied within the library (see the Experimental procedures section) (31, 45, 46). Each library was introduced into the corresponding strain and subjected to negative selection by CRISPR immunity over five consecutive 10-h growth cycles (Fig. 1*C*). After each cycle, the target region of the plasmid library was PCR-amplified and subjected to HTS. Sequences that progressively disappear represent mutations that remain susceptible to either direct interference (*i.e*., lost in both Δ*cas1* and WT) by the original spacer or to priming (*i.e*., lost in WT but not in Δ*cas1*).

Although the plasmid library was designed to strongly represent sequences containing one or two MMs between crRNA and target, it also contained targets with three or more MMs. As expected from previous studies, we found no evidence of direct interference against sequences carrying three or more MMs in the seed sequence (19, 20), as indicated by their relative enrichment throughout the cycles (Fig. S1*A*). Because these mismatched targets should remain stable over time, we normalized our datasets so that the proportion of sequences with three to five MMs remained constant (see the Experimental procedures section) (47). This approach more accurately reflects the depletion of sequences with one or two MMs from the library (Fig. S1*B*).

The normalized frequency of sequences having one or two MMs revealed that 1MM targets were rapidly depleted by the end of cycle 1 in both WT and *Δcas1* strains (Fig. 1*D*). A small fraction of 1MM sequences remained stable in the Δ*cas1* strain, as indicated by their slightly increased frequencies at later time points relative to the WT strain. Notably, 1MM sequences were depleted more in the MS3 *Δcas1* strains relative to the other three Δ*cas1* strains, suggesting that the MS3 crRNA is more tolerant of mutations for direct interference. The normalized frequency of 2MM sequences declined over time in all WT strains but remained enriched in the *Δcas1* strains, indicating that 2MM sequences mostly block direct interference in Δ*cas1* but can support priming in WT strains, similar to previous studies (Fig. 1*D*) (19, 20).

Most of the 1MM sequences were depleted within the first 10-h growth cycle, limiting our ability to analyze the effects of each MM type. We therefore performed the same plasmid library depletion assay using 2-h time points within the time frame of the first 10-h growth cycle. We did not see any significant difference in the loss of 1MM or 2MM sequences between the WT and Δ*cas1* strains across these shorter time points (Fig. 1*E*), consistent with the similar frequency of sequences between the two strains at cycle 1 (Fig. 1*D*). Similarly, when we assessed spacer acquisition through amplification of the CRISPR 2 array for each WT strain bearing the target plasmid library, we observed expanded CRISPRs only following the first 10-h cycle (Fig. S2). Overall, these results indicate that sequences are lost only by direct interference through the first 10 h following induction in our engineered strain and that target loss through priming begins to occur only in the second 10-h cycle.

### Effects of individual MM type and position on CRISPR immunity

**Figure 2:**
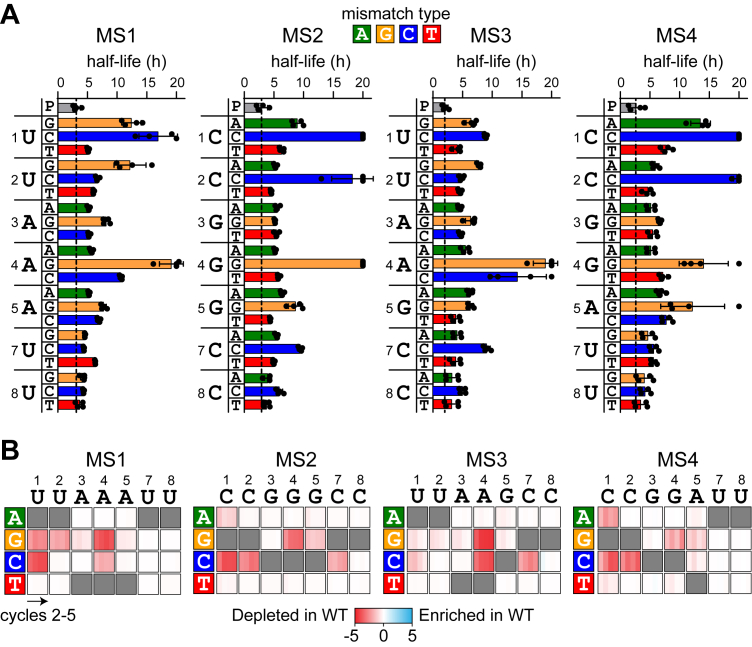
**Influence of mismatch (MM) type and location on direct interference and priming against 1MM seed mutants.***A*, half-lives for sequences containing a single MM at each position of the seed for MS1–MS4.The *dashed line* represents the half-life for the perfect target (P, *gray bar*). The crRNA sequence is listed to the *left* of the axis, and half-lives for the three potential MMs are plotted at each position. MM types are colored as follows: dA = *green*, dG = *orange*, dC = *blue*, and dT = *red*. Half-lives were measured from two replicates from each strain, WT and Δcas1. The bars represent the mean of these half-lives measured from the short time-point data (see Fig. S2 for decay curves). Each data point is plotted as a *black circle*, with error bars representing the standard deviation. Sequences from individual replicates that did not decay sufficiently to fit to the delayed decay model were capped at a half-life of 20 h (see the Experimental procedures section). *B*, heatmaps showing the depletion score for all 1MM target sequences in WT relative to Δ*cas1* strains for MS1–MS4 over 10-h cycles. Low depletion scores (*red*) indicate that sequences were less abundant in WT than in Δ*cas1.* Depletion scores of 0 (*white*) indicate that sequences were equally abundant in WT and Δ*cas1.* Three possible nucleotide substitutions are plotted *vertically* (labeled on *left*) for each position of the crRNA (*horizontal*, labeled on *top*). Depletion scores for cycles 2 to 5 are plotted in each box. *Gray boxes* are shown for complementary target nucleotides. Values represent the average of 2 replicates. crRNA, CRISPR RNA.

As observed in Figure 1*E*, 1MM sequences decayed exponentially following a delay in both the WT and Δ*cas1* strains over a 10-h period. Most individual 1MM sequences also displayed a similar delayed decay (Fig. S3). We fit the normalized frequency of individual 1MM sequences over the 2-h time points to a model that accounts for this time delay prior to exponential delay and calculated half-lives for each sequence (Figs. 2*A* and S3, see the Experimental procedures section). Because the 1MM sequences decayed at similar rates in WT and Δ*cas1* strains (Fig. 1*E*), we included the half-life values for both sets of strains in our analysis.

MMs at positions 1, 22, and 4 of the seed sequence containing C or G in the target sequence caused the longest half-lives, similar to prior studies (19, 20, 39). However, the severity of the effect varied by MM type. In particular, sequences that introduced rC–dC MMs at positions 1 and 22 of MS2 and MS4 were stable and did not decay over the 10-h period (Fig. S3), whereas rC–dC also caused the longest half-lives at position seven. At position 44 of MS1 and MS3, rA–dC was also relatively deleterious. In contrast, while an rU–dC MM was deleterious at position 1 of MS1, rU–dC was relatively tolerated at position 22 of MS1 and at both positions 1 and 22 of MS3 (Fig. 2*A*). For mutations to dG, both rA–dG (MS1 and MS3) and rG–dG (MS2 and MS4) MMs at position 44 were more deleterious than rU–dG at positions 1 and 2 (MS1 and MS3). While rC–dA at position 1 of MS2 and MS4 caused relatively slow decay, rC–dA was tolerated at position 2, as was either rA–dA or rG–dA at position 44 of all four strains. MMs involving dT were generally tolerated at all positions, regardless of the RNA sequence, with rC–dT (MS2 and MS4) causing a slight increase in half-life in comparison to rU–dT (MS1 and MS3) at position 1. Notably, although the four strains contain differing neighboring nucleotides at positions 44 and 5, we observed relatively little difference between the same MMs at these positions (Fig. 2*A*), suggesting that nearest neighbor effects do not substantially influence mutational tolerance within the seed. However, the differences observed at the first two positions of MS1 and MS3 do suggest that overall sequence context may impact mutational tolerance to some degree. Overall, our results indicate that MM type between the crRNA and target DNA has a substantial impact on MM tolerance within the seed sequence.

We next compared how 1MM sequences were depleted in WT *versus Δcas1* strains over the longer, 10-h cycles. As described above, target loss through priming occurs following the first 10-h cycle (Figs. 1*D* and S2) and is expected to result in the loss of 1MM sequences that were stable over the first 10-h cycle (Fig. 2*A*, long half-lives). We calculated depletion scores that measure how the abundance of each 1MM sequence diverges between WT and Δ*cas1* (see the Experimental procedures section). Sequences with half-lives shorter than 10 h (Fig. 2*A*) had depletion scores near 0 (Fig. 2*B*), indicating that they were similarly depleted *via* direct interference in both WT and Δ*cas1* by the end of the first cycle. Sequences with longer half-lives (Fig. 2*A*) had larger negative depletion scores (Fig. 2*B*), indicating that they were depleted in WT but remained abundant in Δ*cas1.* These sequences cannot undergo direct interference but can promote priming, resulting in the loss of these sequences starting in cycle 22 in the WT strains. In general, all 1MM sequences that caused defects in direct interference over the first 10-h cycle promoted priming, with the strongest priming-dependent target loss observed for sequences with the largest direct interference defects (compare Fig. 2, *B* and *A*).

### Combinations of MMs in the seed region promote priming

**Figure 3:**
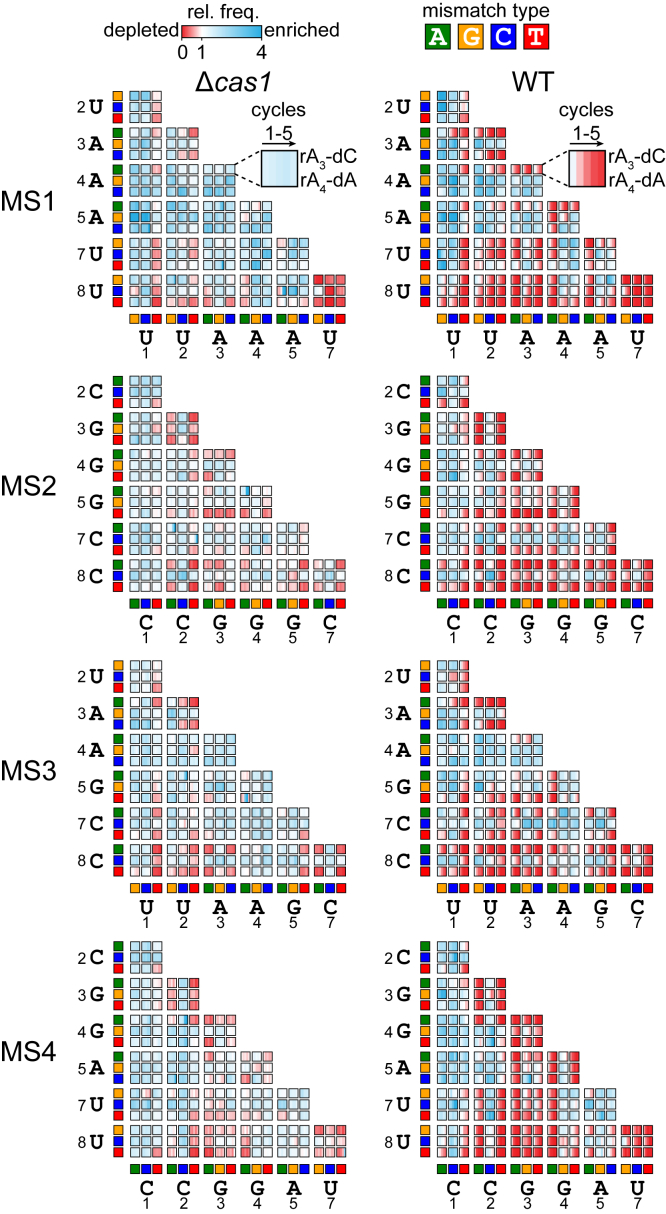
**Comparison of two mismatch (MM) target depletions in WT and Δ*cas1* strains.** Heatmaps showing the relative enrichment (*blue*) and depletion (*red*) of all possible 2MM target sequences over five cycles for all the Δ*cas1* (*left*) and WT (*right*) strains. The relative frequency (rel. freq.) of each 2MM sequence is plotted for the 10-h cycles 1 to 5 (see the Experimental procedures section). The relative frequency values represent the average of n = 2. Each 3 × 3 array represents the nine possible MMs at a given set of two positions. The perfect target sequences are not shown in the heatmap. The MM type is represented by *colored boxes* on the *x*-axis (positions 1–7) and *y*-axis (positions 2–8), with *green* representing dA, *orange* representing dG, *blue* representing dC, and *red* representing dT. An exemplar cell is highlighted for the MS1 Δcas1 and WT samples, showing the combination of rA–dC at position 3 of the crRNA and rA–dA at position 4 of the crRNA. crRNA, CRISPR RNA.

We next examined all target sequences containing two seed MMs (2MM) in Δ*cas1* and WT strains over time (Fig. 3). As predicted from the overall library analysis (Fig. 1, *D* and *E*), only a small number of 2MM sequences were depleted in *Δcas1* strains, whereas many were progressively depleted in WT, showing that these sequences enable priming. The *Δcas1* heatmaps highlight which MM combinations can still support direct interference. Sequences containing 1MM at position 8 in combination with a second MM could often undergo direct interference. This result is expected, as MMs further away from the PAM become progressively less deleterious (20, 39). However, an rC–dC pair at position 8 of MS2 and MS3 is deleterious in combination with most other MMs, whereas several sequences containing rU–dC at position 8 of MS1 and MS3 were depleted in the Δ*cas1* strain, underscoring the relative severity of rC–dC MMs *versus* rU–dC. Conversely, rU–dT at position 1 of MS1 and MS3 is relatively well tolerated in comparison to rC–dT at the same position of MS2 and MS4. Surprisingly, some combinations of MMs at adjacent positions were tolerated. For example, sequences with combinations of rC–dA or rC–dT at position 22 and any MM at position 3 were relatively depleted in MS2 and MS4, whereas sequences with combinations of rU–dC and rU–dT at position 22 and rA–dA and rA–dC at position 3 were relatively depleted in MS1 and MS3. Similar tolerance of adjacent MMs was also observed for some MM combinations at positions 3 and 44 for MS2 and MS4, although rG–dG at position 44 resulted in complete loss of direct interference.

Comparison of the 2MM heatmaps for Δ*cas1* and WT strains indicates that many sequences that were stable in Δ*cas1* were lost *via* priming in WT. However, many sequences also remained stable in the WT strain, indicating sequences that are likely not bound by Cascade. Similar to the 1MM sequences that block direct interference (Fig. 2*A*), the 2MM sequences that block priming are dependent not only on the nucleotide identity of the target mutation but also on the MM type based on the crRNA sequence. For example, rA–dG at position 3 blocks priming for MS1 and MS3 when combined with MMs at positions 1 and 2. In contrast, rG–dG at position 3 can be tolerated for MS2 and MS4, especially in combination with rC–dT at position 2. Nevertheless, for MS4, we did observe tolerance of rG–dG MMs at position 44 and most MMs at position 3, with the exception of a second rG–dG at this position. Thus, while both purine–purine MM types involving dG may be detrimental to Cascade binding, they can be tolerated in some combinations, allowing priming to still occur.

Overall, the combined analysis of 1MM and 2MM sequences that can undergo direct interference and priming, respectively, reveals the importance of the crRNA–DNA MM type in dictating the severity of defects for dG and dC target mutations. While both purine–purine MMs involving dG can be more deleterious than rU–dG, rC–dC is substantially more deleterious than rU–dC or rA–dC.

### The MM type affects Cas8 conformation and target binding dynamics

We next investigated how different MM types impact Cascade conformation and Cas3-mediated degradation rates. Previous work has shown that Cas8 adopts distinct conformations depending on the type of target bound by Cascade (40). The Cas8 N-terminal domain can adopt either a closed or an open conformation, whereas the C-terminal four-helix bundle can adopt either an unlocked or a locked conformation (33, 45, 48, 49). Cas8 adopts a closed and unlocked conformation when unbound to a DNA target (48, 49). When bound to a perfectly matched dsDNA target, Cas8 adopts the closed and locked conformation, which exposes Cas8 residues that contact Cas3, facilitating Cas3 binding and recruitment to the target DNA (29, 33, 34, 42). Cas8 shifts to the open conformation upon ssDNA binding or when mutations are present in the PAM or the seed of dsDNA targets (40, 45), a conformation that is thought to block Cas3 recruitment (29, 34).

**Figure 4:**
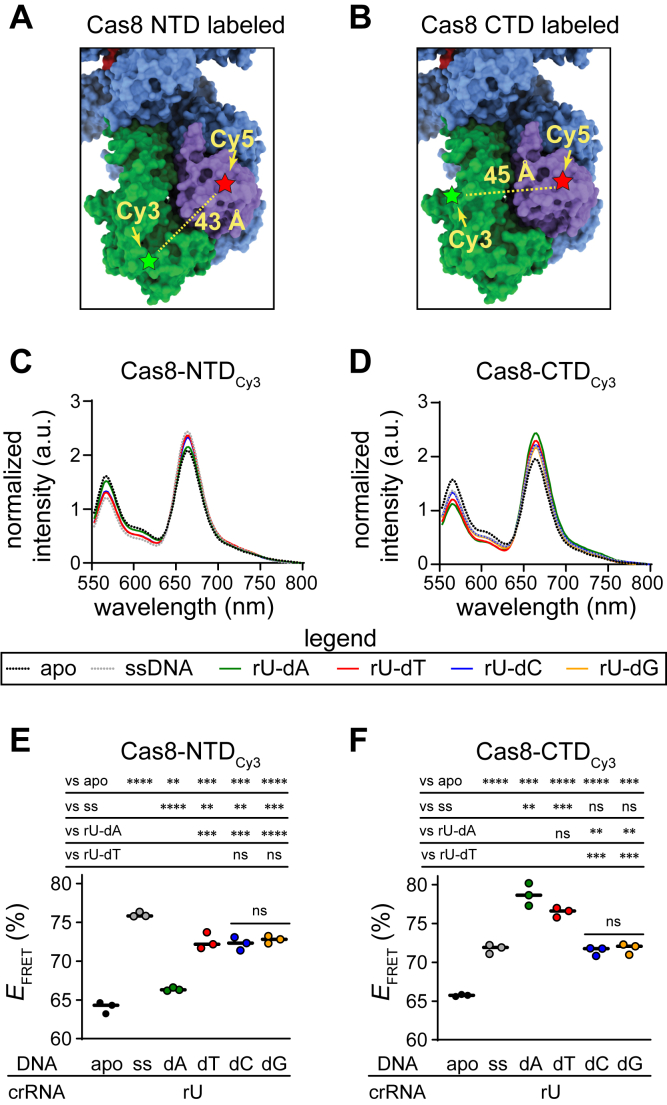
**Measuring Cas8 conformation dependence on mismatch (MM) type using FRET.** The position of the Cy3 label (*A*) on the N-terminal domain (NTD) of Cas8 (Cys 69) or (*B*) on the C-terminal domain (CTD) of Cas8 (Cys 376); Cy5 is on Cas5 (Cys 169). *C* and *D*, normalized FRET intensity for NTD- and CTD-labeled Cascade bound to ssDNA, perfect target (rU–dA), rU–dT MM, rU–dC MM, and rU–dG MM. MMs were located at the first position of the target. *D*, the chromatograms for ssDNA, rU–dC, and rU–dG overlap with one another. *E* and *F*, FRET efficiency for NTD- and CTD-labeled Cascade bound to different targets. FRET efficiency values for three technical replicates are shown as *circles*, and the mean is represented as a *black line*. *p* Values from unpaired, two-tailed *t* tests are reported above the graph. ∗∗*p* < 0.01; ∗∗∗*p* < 0.001; ∗∗∗∗*p* < 0.0001; and ns = *p* > 0.05. Individual graphs and controls are shown in Figure S4. ns, not significant.

To examine how different MM types influence Cas8 conformation, we performed a previously developed FRET assay (40) using Cascade bearing the MS1 crRNA. In this assay, the N-terminal domain or C-terminal domain of Cas8 is labeled with Cy3, and the Cas5 subunit is labeled with Cy5 (Fig. 4, *A and B*). The N-terminal domain of Cas8 moves toward Cas5 when it adopts the open conformation, causing an increase in FRET signal (Fig. 4*A*). The C-terminal domain of Cas8 moves toward Cas5 when it adopts the locked conformation, causing an increase in FRET signal (Fig. 4*B*).

We measured Cas8 conformation when Cascade was bound to dsDNA targets containing either a perfectly matched DNA (rU–dA at position 1) or targets containing all three MMs at position 1. A ssDNA target was also included as a positive control for the open conformation. The perfectly matched DNA and ssDNA controls produced changes in FRET efficiency in comparison to *apo*-Cascade similar to changes we observed previously using a different crRNA (40). When the N-terminal domain of Cas8 was labeled, all three mismatched targets had similar FRET efficiencies (Figs. 4, *C* and *E*, S4*A*). FRET efficiencies for the mismatched targets were between the perfect dsDNA and ssDNA controls, suggesting that the three mismatched targets induce the open conformation to a similar degree that exceeds the perfect dsDNA target but not to the same degree as the ssDNA target. In contrast, when the C-terminal domain of Cas8 was labeled, we observed similar FRET efficiencies for the perfect target and the rU–dT MM, whereas rU–dC and rU–dG had FRET efficiencies more similar to the ssDNA control (Figs. 4, *D* and *F*, S4*B*). These findings suggest that Cas8 adopts an alternative conformational ensemble when MS1-Cascade binds to the dT mismatched target, relative to the dC and dG targets.

**Figure 5:**
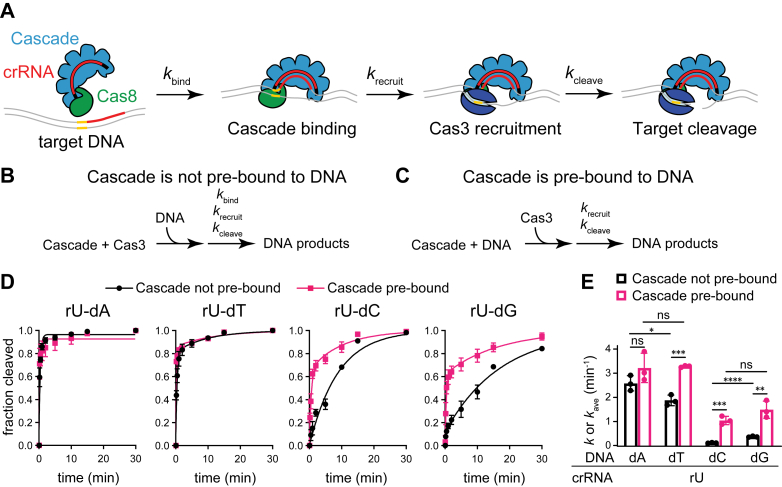
**Measuring the effects of mismatch type on the rate of target binding and degradation.***A*, kinetic steps that determine the rate of target degradation by Cascade–Cas3 complex. The rates of target binding (represented by *k*_bind_), Cas3 recruitment (*k*_recruit_), and target cleavage (*k*_cleave_) all contribute to the overall rate of target degradation. *B* and *C*, schematic view of experimental design. *B*, when Cascade and Cas3 are mixed together prior to the addition of DNA, target degradation is dependent on the rate of target binding, Cas3 recruitment, and target cleavage. *C*, when Cascade is incubated with DNA to allow prebinding prior to the addition of Cas3, the rate of target degradation is dependent only on the rate of Cas3 recruitment and target cleavage. *D*, fraction cleaved over time for Cascade is not prebound to DNA (experimental design shown in *B*), and Cascade is prebound to DNA (experimental design shown in *C*). Data points are the average of three technical replicates, and standard deviation is shown as error bars. The data were fit to a double-exponential rate equation, with the exception of the perfect target (rU–dA) in both conditions and the rU–dC target when Cascade was not prebound, which fit better to a single-exponential rate equation (see the Experimental procedures section). *E*, observed rate constants (*k*) or average observed rate constant (*k*_avg_) values for Cascade bound to different targets. For targets that fit best to a double exponential, *k*_avg_ was determined by averaging the fitted rate constants for the fast and slow phases, with the exception of the rU–dA target in both conditions and the rU–dC target when Cascade was not prebound, for which a single *k* value was determined (see the Experimental procedures section). The average of three technical replicates is shown, with error bars representing standard deviation. *p* Values based on unpaired, two-tailed *t* tests are indicated for each target between assays and for rU–dA *versus* rU–dT or rU–dC *versus* rU–dG within each assay. Additional *p* values are omitted from the graph for clarity and are reported in Table S5. ∗*p* < 0.05; ∗∗*p* < 0.01; ∗∗∗*p* < 0.001; ∗∗∗∗*p* < 0.0001; and ns = >0.05. ns, not significant.

To determine whether the conformational differences induced by the dC and dG mismatched target impact Cas3-dependent target degradation, we performed Cascade–Cas3 cleavage assays against plasmid targets bearing either the perfect target or the three mismatched targets at position 1 (Fig. S5). In a simplified kinetic model, the overall rate of target degradation is based on the rate of target binding by Cascade (*k*_bind_), Cas3 recruitment to Cascade (*k*_recruit_), and target cleavage by Cas3 (*k*_cleave_) (Fig. 5*A*). MMs at position 1 are expected to decrease the rate of target binding (*k*_bind_), which may mask the effects of Cas8 conformation on the rate of Cas3 recruitment and subsequent steps (*k*_recruit_ and *k*_cleave_). We therefore performed cleavage assays in two different ways. In the first assay, Cascade and Cas3 were premixed prior to the addition of the DNA target, resulting in cleavage rates that are dependent on all three phases of our kinetic model (Fig. 5*B*). In the second assay, Cascade and the target were incubated together, allowing Cascade to prebind the DNA prior to the addition of Cas3 (Figs. 5*C* and S5*A*). This experimental design eliminates the rate of Cascade–target binding from the kinetic model and should more directly inform on the rate at which Cas8 recruits Cas3.

We observed that the target degradation reactions were biphasic for most targets and fit well to a double-exponential rate equation, with the exception of the perfect (rU–dA) target in both assays and the rU–dC target when Cascade was not prebound to the DNA (Fig. 5*D*). To compare *k* values, we averaged the rate constants measured for the slow and fast phases for cleavage data that were fit to a double-exponential model to account for the biphasic nature of the reaction (see the Experimental procedures section) (Fig. 5*E*). We observed similar rates for the rU–dA and rU–dT targets, both of which were degraded much more rapidly than targets that introduce an rU–dC or rU–dG MM at position 1 for both types of assays (Fig. 5*D*). When Cascade was not prebound to the target, the rU–dT MM caused a slight decrease in the rate of cleavage (∼1.4x lower than the perfect target, *p* = 0.03 based on a two-tailed, unpaired *t* test). However, when Cascade was prebound to the target, we observed no significant difference in the rate of cleavage for the perfect target and the rU–dT mismatched target. These results suggest that the rU–dT target has a small effect on the rate of target binding, but that Cas3 recruitment and cleavage activity are unaffected by the differences in Cas8 conformation induced by the rU–dT MM at position 1.

For the other two mismatched targets, when Cascade was prebound to the DNA, we observed a two to three times decrease in the rate of target degradation compared with the perfect target and the rU–dT mismatched target (Fig. 5*E*). When Cascade was not prebound to the DNA, this difference was substantially larger, with the rU–dC target causing a 23x decrease and the rU–dG target causing a 7x decrease compared with the perfect target. Collectively, these results indicate that both the rU–dC and rU–dG MMs decrease the rate of Cas3 recruitment to a similar degree, whereas the rU–dC causes a larger defect in the rate of target binding in comparison to the rU–dG MM.

## Discussion

In this study, we have systematically investigated the effect of the crRNA seed sequence on CRISPR interference and priming in the type I–E CRISPR–Cas immune system. Our results confirm that depending on the position and type of MMs, CRISPR immunity can tolerate up to two MMs in the seed region and promote priming. Similar to previous studies, we observed that dC and dG mutations in the target strongly inhibit direct interference (20, 38, 39), especially when located at positions 1, 2 and 4 of the seed. However, our findings also implicate the crRNA nucleotide in mutational tolerance. For dC mutations, rU and rA in the crRNA provided greater tolerance than rC, whereas for dG mutations, rU provided greater tolerance than rG or rA.

Previous structural modeling of multiple MM types provided insight into the impact of the crRNA–DNA MM type on Cascade–target binding (38). In particular, rA–dG creates a clash because of the bulkiness of purine–purine base pairing, whereas rU–dG creates a clash with the thumb region of the Cas7 backbone subunit. Although dC MMs were better accommodated within these structural models, we note that rC–dC is among the most deleterious MM type in our plasmid loss experiments. Similarly, we observed that an rU–dC MM causes a larger binding defect than an rU–dG MM for the MS1 crRNA (Fig. 5*E*), consistent with the rate of direct interference that we observed in cells (Fig. 2*A*). Thus, it is unclear whether Cascade accommodation of mismatched target sequences alone is sufficient to explain the effects of certain types of MMs. It is tempting to speculate that dC and dG MMs are more deleterious to DNA unwinding by Cascade. However, while it is important to consider the overall energy of target unwinding, it is also essential to consider how the MM type that is introduced affects Cascade–target binding. Certain MMs could potentially enable the formation of a more stable R-loop than other MM types, which is required for Cas3 recruitment (29, 34, 35).

An additional consideration is the effect of MM type on Cascade conformation. The conformation of the Cas8 subunit is sensitive to mutations in or near the PAM (40, 41), and the conformation of this subunit is also important for ensuring efficient Cas3 recruitment and target degradation (29, 33, 34, 42). We find that the rU–dT MM that is best tolerated for direct interference at position 1 of the MS1 target (Fig. 2*A*) adopts a different Cas8 conformation than rU–dC or rU–dC (Fig. 4*F*), resulting in more rapid cleavage of the target when Cascade is prebound to the target DNA (Fig. 5*E*). These collective results strongly suggest that MM type may impact the rate of interference not only by impacting the rate of target binding but also by reducing the rate of Cas3 recruitment following target binding.

Overall, our study provides initial insight into the impact of MM type on type I–E interference and priming activities. An important limitation of our and previous studies is the difficulty of investigating all possible MM types at all positions (19, 20, 37, 39). Although our dataset covered four distinct crRNA sequences, it still lacks several MM types (*e.g.*, rG or rA MMs at the first two positions of the seed). Future studies may focus on the impact of other MM types as well as the importance of MM type in the context of phage escape.

## Experimental procedures

### Bacterial strains

Primers for *E. coli* engineering are listed in Table S1, and strains used in the study are listed in Table S2. All strains used in this study originated from the BW25113 strain (44, 50), which originally contained 18 spacers between two CRISPR arrays (1 and 2). We modified each CRISPR to only contain a single spacer (1.1 and 2.1), as previously described (19). The native *cas3* and *cas8* promoters were replaced with *tac* and *araBp8*, respectively, using λ-Red recombinase (50). Four different designed seed sequences (MS1: 5′-TTAAAATT-3′, MS2: 5′-CCGGGGCC-3′, MS3: 5′-TTAAGCCC-3′, and MS4: 5′-CCGGATTT-3′) were introduced into the CRISPR 22 locus using a previously developed method for scarless λ red chromosomal manipulation based on a kanamycin resistance marker and mPheS (modified *E. coli* phenylalanine tRNA synthetase) for counter selection (51). Outside the seed sequence, the CRISPR 22 spacer 1 sequence was retained. Following replacement of the seed sequences, a Δ*cas1* strain was constructed for MS1–MS4 using λ-Red recombination (50). All genetic changes were initially verified by PCR amplification of the altered genomic region and Sanger sequencing (Eurofins Genomics). The MS1 and MS1 Δ*cas1* strain genomes were also sequenced using nanopore sequencing (Plasmidsaurus). The exact genotypes of these strains are provided in Table S1.

### Generation of seed library

To generate partially randomized seed sequence libraries, four different single-stranded oligonucleotides with 8% doping frequency were designed and synthesized (Integrated DNA Technologies). Here, each position in the seed sequence has a 76% chance of having the correct nucleotide that matches the crRNA (47). The crRNA of Cascade does not base pair with every sixth nucleotide of the protospacer target strand (20, 31, 39, 45, 46). Hence, we utilized this unique sixth position to serve as another barcode for analyzing the high-throughput sequence data (see below), with each library having a different nucleotide at the sixth position (MS1 = A; MS2 = G; MS3 = C; and MS4 = T). Oligonucleotides were assembled with pACYC-GFP (52) using the NEBuilder HiFi DNA Assembly Cloning Kit, and the products were transformed into NEB Stable competent cells as per the manufacturer’s protocol. Around 50,000 colonies were generated for each library on LB-agar plates supplemented with 34 μg/ml chloramphenicol. The colonies were resuspended with 50 ml of LB supplemented with chloramphenicol (34 μg/ml) and grown overnight at 37 °C with shaking at 180 rpm. The plasmid libraries were then extracted using a Qiagen Miniprep DNA Purification kit. Plasmid selection and HTS were performed for two biological replicates.

### Plasmid library depletion assay

Each library was transformed into the corresponding WT and Δ*cas1* strains. During the recovery stage, the cells were then directly cultured in 50 ml of LB with chloramphenicol and grown overnight at 37 °C with shaking at 200 rpm. This culture was used as cycle 0. The next day, 20 μl of each culture was inoculated into 2 ml fresh LB supplemented with 2 mM IPTG, 20 mM arabinose, and no antibiotics. The culture was grown for 10 h, with time points taken every 2 h, or for five 10-h cycles, with time points taken for each cycle and subculturing in fresh media with inducers at the end of each cycle. The genomic DNA from each time point or cycle was extracted for use as templates for PCR. For each time point, new spacer acquisition was detected *via* PCR of the CRISPR arrays within the genomic DNA using Taq DNA polymerase. Newly acquired spacers in CRISPR 22 were detected *via* PCR using primers listed in Table S1 that anneal to the leader sequence and the constant region of the first spacer sequence.

### Preparation of samples for HTS

To prepare samples for Illumina sequencing, the original library miniprep and genomic DNA from each time point/cycle were used as templates for PCR amplification of the target region using Q5 High Fidelity DNA Polymerase (New England Biolabs) with Nextera adapters (Table S1). The 132 bp product was then analyzed by 2% agarose gel electrophoresis and purified by the QIAquick PCR Purification Kit (Qiagen). These products were then amplified by PCR using Q5 High-Fidelity DNA Polymerase (New England Biolabs) using a pair of primers containing unique barcodes to differentiate between libraries and replicates. The 224 bp product was analyzed by 2% agarose gel electrophoresis and purified by the QIAquick PCR Purification Kit. The samples were mixed, and equal amounts were pooled based on their absorbance reading. Samples were analyzed using an Agilent 2100 Bioanalyzer to determine the size and purity and submitted to Admera Health or the Iowa State DNA Facility for Illumina MiSeq analysis with paired-end reads of 75 or 150 cycles.

### HTS analysis

Python scripts used for analysis of plasmid library sequencing data are available at github.com/sashital/typeIE_seed_sequence. The scripts were written in collaboration with ChatGPT (OpenAI, January 2026 model) and were extensively validated.

Seed sequences were extracted based on matching of the constant sequences upstream and downstream of the seed and the presence of a barcoded sixth position described above. The number of reads for each unique seed sequence for each cycle of a given replicate for a given strain was output as a tab-separated file.

Next, the MM frequency was calculated for each number of MM (*n*) at each cycle (*c*) (Fig. S1*A*).

$${\text{MM}\;\text{frequency}}_{nc}=\frac{\text{Count}\;\text{of}\;\text{sequences}\;\text{with}\;n\;\text{mismatches}\;\text{in}\;\text{cycle}\;c}{\text{Total}\;\text{counts}\;\text{in}\;\text{cycle}\;c}$$

To adjust the abundance of different enriched sequences, a normalization factor (NF) was calculated by averaging the MM frequency of sequences with three, four, and five MMs from each cycle (*c* = 1, 2, 3, 4 or 5) and dividing the average 3MM to 5MM frequency at cycle 0 by the value at each individual cycle.

$${\text{NF}}_{c}=\frac{{\text{Average}\;3-5\;\text{MM}\;\text{frequency}}_{c=0}\;}{{\text{Average}\;3-5\;\text{MM}\;\text{frequency}}_{c}}$$

Each NF was multiplied by the frequency from the original MM distribution to generate a normalized MM frequency (Fig. S1*B*).

$${\text{Normalized}\;\text{MM}\;\text{frequency}}_{nc}={\text{NF}}_{c}*\;{\text{MM}\;\text{frequency}}_{nc}$$

For 1MM and 2MM depletion curves (Fig. 1, *D* and *E*), the normalized MM frequency for each cycle was divided by the MM frequency at cycle 0 prior to plotting *versus* cycle number or time point.

The frequency of individual 1MM or 2MM target sequences was measured by dividing the number of counts for sequences with the specific number of MMs by the total count at a given 10-h cycle or 2-h time point.

$${\text{Frequency}}_{c}=\frac{{\text{Count}\;\text{of}\;\text{individual}\;\text{sequence}}_{c}\;}{\;{\text{Total}\;\text{count}\;}_{c}}$$

The frequency relative to the control was determined by dividing the frequency at a given cycle by the frequency of the same sequence at cycle 0.

$${\text{Relative}\;\text{frequency}}_{c}=\frac{{\text{Frequency}}_{c}}{{\text{Frequency}}_{c=0}}$$

For data that were fit to a delayed exponential decay model (see below), the original library miniprep was used as the control, as we observed a small amount of target loss during cycle 0, likely because of leaky expression of the *cas* operon.

The normalized frequency was determined by multiplying the relative frequency for a given sequence at a given cycle by the NF for that cycle.

$${\text{Normalized}\;\text{frequency}}_{c}={\text{relative}\;\text{frequency}}_{c}\;\times \;{\text{NF}}_{c}$$

To determine the half-lives shown in Figure 2*A*, normalized frequency values for 2-h time points were fit using a delayed exponential decay model in GraphPad Prism 10 (GraphPad Software Inc.) (individual plots are shown in Fig. S3). The model assumes an initial plateau followed by first-order decay:

$$y=\left\{ \begin{array}{c} 1,t\;\leq \;t_{d}\\ e^{-k\left(t-t_{d}\right)},t\;>\;t_{d} \end{array}\right.$$

where *y* is the normalized frequency at a given time point *t*, *k* is the decay rate constant, and *t*_d_ is the delay time before decay begins. The initial value was fixed at 1, and the decay plateau was fixed at 0. The half-life was determined by adding the delay time to the half-life calculated using the decay rate constant:

$$t_{50}=t_{d}+\ln\left(2\right)/k$$

Fits were performed independently for each replicate for both the WT and *cas1* deletion strains. Sequences that did not decay sufficiently to obtain a good fit were capped at a half-life of 20 h.

For the comparison of 1MM sequence depletion between the WT and *cas1* deletion strains across 10-h cycles in Figure 2*B*, the normalized frequency of each 1MM sequence at each cycle from the WT and Δ*cas1* strains was calculated, and the difference was assessed by the depletion score (*Z*).

$$Z=\frac{{\text{Normalized}\;\text{frequency}}_{WT}-{normalized\;frequency}_{\Delta \text{cas}1}}{\sigma \;}$$

where σ is the standard deviation of all 1MM sequences in the WT and Δ*cas1* strain for a given replicate at a given cycle. Negative values indicate the depletion of the 1MM sequences in the WT but not Δ*cas1*, and values close to 0 represent sequences that were depleted in both WT and Δ*cas1*.

For the heatmaps in Figure 3, the relative frequency of all 2MM sequences was plotted for cycles 1 to 5. In the development of the heatmaps, we plotted both relative frequency and normalized frequency. The relative frequency plots without normalization to the 3–5MM sequences provided the best contrast between sequences that are tolerated and sequences that cause defects in interference and/or priming and are used in Figure 3. The average of two replicates is plotted for each sequence at each cycle.

### Plasmids used for protein expression and target cleavage

All plasmids used in this study are listed in Table S3 and were verified by Sanger sequencing and whole plasmid sequencing (Eurofins Genomics). Plasmids used for expression of Cas3 and WT or minimal cysteine variants of Cascade without the Cas8 subunit (hereafter Cascade_-8_) and Cas8 are previously described (2, 19, 40). For expression of Cascade bearing the MS1 spacer, a CRISPR array containing eight repeats and seven MS1 spacers in pET-52b(+) was synthesized by GeneScript and subcloned into pRSF under control of a T7 promoter with a *lac* operator by Gibson Assembly. MS1 target plasmids were constructed using pUC19. The target sequences (Table S1) were inserted between BamHI and EcoRI cut sites using restriction cloning. Target plasmids were purified using the Qiagen Plasmid Midi Kit.

### Protein purification

All proteins were expressed in BL21(DE3) cells. Overnight cultures were used to inoculate large-scale cultures for protein expression. All cultures were grown to 0.5 at an absorbance at 600 nm at 37 °C and induced overnight at 20 °C with 0.5 mM IPTG.

For WT Cascade_-8_, cells were lysed in lysis buffer 1 (50 mM sodium phosphate dibasic [pH 8.0], 500 mM sodium chloride, 5% glycerol, 10 mM imidazole [pH 8.0], cOmplete, EDTA-free Protease Inhibitor Cocktail, 1 mM DTT) using an Avestin homogenizer. Cell debris was removed by centrifuging the cell lysate at 19,000 rpm for 30 min. The supernatant was collected and run through HisPur nickel–nitrilotriacetic acid (Ni–NTA) affinity resin in lysis buffer 1, washed with wash buffer 1 (50 mM sodium phosphate dibasic [pH 8.0], 500 mM sodium chloride, 5% glycerol, 25 mM imidazole [pH 8.0], and 1 mM DTT) and eluted with elution buffer (50 mM sodium phosphate dibasic [pH 8.0], 500 mM sodium chloride, 5% glycerol, 250 mM imidazole [pH 8.0], and 1 mM DTT). The eluted proteins were cleaved by tobacco etch virus protease overnight at 4 °C to remove the His_6_-tag while dialyzing against dialysis buffer (50 mM sodium phosphate dibasic [pH 8.0], 500 mM sodium chloride, 5% glycerol, and 2 mM DTT). The cleaved proteins were flowed through a Ni–NTA column to remove uncleaved protein, concentrated to 1 ml, and purified on a Superdex 200 column in size exclusion buffer 1 (20 mM Tris [pH 7.5], 100 mM NaCl, 5% glycerol, and 1 mM DTT). Minimal cysteine Cascade_-8_ with K169C Cas5 (hereafter K169C-Cascade_-8_) was purified in the same way, except using Tris(2-carboxylethyl)phosphine (TCEP) instead of DTT.

Cas3 was purified as above with the following differences in buffers. Cells were lysed in the lysis buffer 2 (20 mM Tris–HCl [pH 8.0], 100 mM sodium chloride, 1% glycerol, 10 mM imidazole [pH 8.0], cOmplete, EDTA-free Protease Inhibitor Cocktail, and 2 mM DTT) using an Avestin homogenizer. The supernatant after centrifugation was run through HisPur Ni–NTA affinity resin in lysis buffer 2, washed with wash buffer 2 (1 M NaCl, 25 mM imidazole [pH 8.0], 10% glycerol, and 2 mM DTT) and then washed with wash buffer 1 and eluted with elution buffer. Size exclusion was performed in size-exclusion buffer 2 (20 mM Tris–HCl [pH 8.0], 200 mM NaCl, 5% glycerol, and 2 mM DTT).

WT Cas8 was purified *via* HisPur Ni–NTA affinity resin as described above for Cascade_-8_. The eluted proteins were cleaved with tobacco etch virus protease overnight at 4 °C to remove the MBP-His_6_-tag while dialyzing against buffer A (50 mM Hepes [pH 7.0], 50 mM sodium chloride, 5% glycerol, and 2 mM DTT). Cleaved samples were applied to a HiTrap HP-SP (GE Healthcare Life Sciences) ion exchange column equilibrated with buffer A to separate the MBP-His_6_ tag from Cas8. The column was washed with 10% buffer B (50 mM Hepes, pH 7.0, 1 M NaCl, 5% glycerol, and 1 mM DTT). Bound proteins were eluted by a gradient from 10% to 50% buffer B. The sample was concentrated to 1 ml and applied to Superdex 200 column in size-exclusion buffer 1 (20 mM Tris [pH 7.5], 100 mM NaCl, 5% glycerol, and 1 mM DTT). Minimal cysteine H69C Cas8 (hereafter H69C-Cas8) and minimal cysteine N376C Cas8 (hereafter N376C-Cas8) were purified in the same way, except using TCEP instead of DTT.

### Protein labeling for FRET assays

Cy3-maleimide and Cy5-maleimide (Lumiprobe) were dissolved in 50% dimethyl sulfoxide to 4 mM. All labeling reactions were performed in the labeling/cleavage buffer (50 mM Hepes NaOH [pH 7.5], 100 mM KCl, 5% glycerol, and 1 mM TCEP). H69C-Cas8 was used to label the N-terminal domain of Cas8, and N376C-Cas8 was used to label the C-terminal domain of Cas8. Each Cas8 variant was labeled with the Cy3 donor fluorophore, and minimal cysteine Cascade_-8_ was labeled with the Cy5 acceptor fluorophore. A Cas8 variant or K169C-Cascade_-8_ (20 μM) was mixed with 200 μM Cy3 or 200 μM Cy5, respectively, with a final dimethyl sulfoxide concentration of 5%. Reactions were incubated in the dark at 4 °C for 1 h. Reactions were quenched by adding 10 mM DTT, and labeled proteins were separated from free dye using a Spin-X UF 10K molecular weight cutoff concentrator (Corning). For each set of experiments, proteins were labeled just prior to the experiment.

### DNA substrates for FRET assays

DNA substrates for FRET assays (Table S1) were chemically synthesized by Integrated DNA Technologies and purified by denaturing gel electrophoresis using 12% polyacrylamide with 8 M urea in 1*X* Tris–boric acid–EDTA buffer. DNA was excised from the gel and recovered by crushing the gel pieces and soaking in ddH_2_O overnight at 4 °C. The gel pieces were removed by filtration using Costar Spin-X centrifuge tube filters (Corning) and ethanol precipitated. The dried pellet was resuspended in ddH_2_O, and concentration was determined using an absorbance at 260 nm measured on a Nanodrop (Thermo Fisher Scientific). The nontarget strand was annealed with the target strand at a 1.2:1 M ratio in labeling/cleavage buffer by heating at 95 °C for 5 min and cooling to room temperature. To ensure complete binding, the nontarget strand was truncated ten-nt after the PAM sequence, as previously described (40).

### FRET sample preparation, measurement, and analysis

All fluorescence measurements were conducted in labeling buffer. Each Cas8-Cy3 variant and 100 nM K169C-Cascade_-8_-Cy5 (150 nM) were mixed in 100 μl reactions and incubated at room temperature for 30 min to form the Cascade complex. Cascade complex was mixed with the DNA target at a 1:1.5 ratio and incubated at room temperature for 30 min. For each sample, control experiments were performed to detect donor or acceptor fluorescence in the absence of their corresponding FRET pair. These samples were prepared identically to FRET samples, but unlabeled K169C-Cascade_-8_ (donor alone) or a Cas8 variant (acceptor alone) was substituted to enable DNA binding but prevent FRET.

After incubation, the fluorescence of reactions was detected at 25 °C using black 96-well plates (Thermo Fisher Scientific) and a Tecan Spark Multimode Microplate reader. The protocol was set to ten flashes with 100% gain, Z-position was set to 20000 μm, settle time was set to 5 ms with 10 μs lag time, and 50 μs integration time. The excitation filter was set to 485 (20) nm, and the emission wavelength was measured from 553 nm to 800 nm, with a 5 nm bandwidth with 2 nm increments. Fluorescence measurements were performed in triplicate. Fluorescence intensity spectra shown in Figure 4, *C* and *D* were normalized and smoothed using GraphPad Prism. The raw fluorescence intensity spectra, including controls, are shown in Figure S4.

Raw data was analyzed using a previously described method (40). *E*_*FRET*_ was determined by calculating donor fluorescence quenching (53):

$$E_{FRET}=1-\left[\left(\text{IDA}-\text{IA}\right)/\text{ID}\right]$$

where IDA is the donor fluorescence intensity in the presence of the acceptor, IA is the acceptor fluorescence in the absence of the donor, and ID is the donor fluorescence intensity in the absence of the acceptor. All values were calculated by integrating the area under the peaks from 550 to 620 nm, using GraphPad Prism. The change in *E*_*FRET*_ (Δ *E*_*FRET*_) was calculated by subtracting the *E*_*FRET*_ value for apo Cascade from Cascade bound to targets. The average of three replicates was determined, and standard deviation was propagated for subtraction to determine error.

### Plasmid degradation assays

All reactions were performed in labeling buffer, with the addition of 10 μM CoCl_2_, 10 mM MgCl_2_, and 2 mM ATP (hereafter cleavage buffer). Reactions were performed at room temperature to allow measurement of degradation rates for the perfect target, which occurs too rapidly to perform kinetic analysis at 37 °C. Plasmid degradation was performed through two different types of assays. For the experiments in which Cascade was not prebound to DNA, 150 nM WT Cas8, 100 nM WT Cascade_-8,_ and 500 nM Cas3 were premixed. Then, 6 nM target plasmid was added, and the reaction was incubated at room temperature. At each time point, an aliquot was removed, and the reaction was terminated by the addition of phenol–chloroform in a 1:1 ratio. For experiments in which Cascade was prebound to DNA, to eliminate the effects of binding defects on the rate of target degradation, 150 nM WT Cas8 and 100 nM WT Cascade_-8_ were mixed with 6 nM plasmid. The binding reactions were incubated for 30 min at room temperature. Then, 500 nM Cas3 was added, and aliquots of the reaction were terminated by addition of phenol–chloroform for each time point in a 1:1 ratio. For both experiments, the aqueous layer was extracted and mixed with a 2X gel loading solution in a 1:1 ratio and analyzed by electrophoresis on a 1% agarose gel with poststaining using SYBR Safe Gel Stain (Thermo Fisher Scientific).

To ensure that Cascade bound to all target plasmids, binding reactions were prepared identically to cleavage assays where Cascade was prebound to DNA. After incubation, samples were mixed with 10% glycerol in a 1:1 ratio and analyzed by electrophoresis on a 0.7% agarose gel. The gel was run at 20 V for 30 h at 4 °C and visualized *via* poststaining using 1*X* SYBR Safe Gel Stain (Thermo Fisher Scientific) (Fig. S5*A*).

The intensity of DNA bands was quantified by densitometry using ImageJ software (https://www.nature.com/articles/nmeth.2089). Cas3 initially nicks the DNA, followed by degradation, resulting in an initial slow-migrating relaxed band and eventual linearization and smearing of the DNA throughout the lane (Fig. S5, *B–E*). The intensities of the nicked, linear, negatively supercoiled, and degraded DNA were measured separately. To calculate the fraction cleaved, the product intensity (including nicked, linearized, and degraded DNA) was divided by the total DNA (including supercoiled, nicked, linearized, and degraded DNA). For conditions where a nicked band appeared prior to cleavage initiation, the intensity of the nicked DNA at time point 0 was subtracted from the intensity of the nicked DNA at a given time point prior to determining the fraction cleaved. Each measurement was performed in triplicate.

For fitting fraction cleaved *versus* time, most of the rate curves fit best to a double-exponential rate equation

$$y=A_{fast}\times \left(1-e^{\left(-k_{fast}\;\times \;x\right)}\right)+A_{slow}\;\times \;\left(1-e^{\left(-k_{slow}\;\times \;x\right)}\right)$$

where *A*_fast_ and *A*_slow_ are the amplitudes of the fast and slow phase, respectively, and *k*_fast_ and *k*_slow_ are rate constants for the fast and slow phase, respectively. All rate curves were fit to this equation with the exception of the perfect target (rU–dA) for both conditions and the rU–dC target when Cascade was not prebound to the target, which fit better to a single-exponential rate equation

$$y=A\times \left({1-e}^{\left(-k\;\times \;x\right)}\right)$$

The data were fit in GraphPad Prism. The fits of average data from the three replicates are shown in Figure 5*D*.

To summarize rate constants measured using the double-exponential rate equation, *k*_ave_ was calculated by using the following formula:

$$k_{\text{ave}}=\left(A_{\text{fast}}\times k_{\text{fast}}\right)+\left(A_{\text{slow}}\times k_{\text{slow}}\right)$$

For the targets where a single-exponential rate equation was used, the *k* value determined from the fit is reported. Values for *k* or *k*_ave_ were determined for each replicate, and average values were plotted in Figure 5*E*, with error representing the standard deviation of the three replicates. The underlying values for calculating *k*_ave_ are provided in Table S4.

### Data availability

All Python scripts are published in a GitHub repository (https://github.com/sashital-lab/typeIE_seed_sequence) and can be accessed at https://doi.org/10.5281/zenodo.18499583. The underlying fastq files are deposited at https://doi.org/10.17632/d573m6gpfs.1. The data underlying biochemistry experiments shown in Figures 4 and 5 are provided or represented in the supporting information.

## Supporting information

This article contains supporting information (2, 19, 32, 40, 44).

## Conflict of interest

The authors declare that they have no conflicts of interest with the contents of this article.
